# Service-learning using the BOPPPS model in TCM nursing education: a mixed-methods study

**DOI:** 10.3389/fmed.2026.1859149

**Published:** 2026-09-09

**Authors:** Shujie Hao, Haixia Xu, Zheyuan Xia, Yue Wang, Huiling Zhang, Ting Wang, Yamei Yuan, Hui Shi

**Affiliations:** 1 School of Nursing, Anhui University of Chinese Medicine, Hefei, Anhui, China; 2 Key Laboratory of Geriatric Nursing and Health, Anhui University of Chinese Medicine, Hefei, Anhui, China; 3 Department of Nursing, The First Affiliated Hospital of Anhui University of Chinese Medicine, Hefei, Anhui, China; 4 Department of Nursing, The Second Affiliated Hospital of Anhui University of Chinese Medicine, Hefei, Anhui, China

**Keywords:** BOPPPS model, nursing education, service-learning, traditional Chinese medicine nursing, undergraduate nursing students

## Abstract

**Background:**

Service-learning is increasingly used in nursing education to connect classroom learning with authentic practice and community engagement. However, evidence on how instructional models can optimize service-learning in nursing education remains limited. This study aimed to examine the educational effects of service-learning using the BOPPPS model in an undergraduate traditional Chinese medicine (TCM) nursing course.

**Methods:**

This mixed-methods study included a quasi-experimental quantitative component and a qualitative component using thematic analysis. Ninety-six undergraduate nursing students participated, including 49 in the experimental group and 47 in the control group. The experimental group received service-learning using the BOPPPS model, whereas the control group received conventional teaching. Quantitative outcomes included course scores, service-learning abilities, and course evaluation scores. Qualitative data were collected through final reflective reports and analyzed to explore students' learning experiences.

**Results:**

Compared with the control group, the experimental group achieved better academic performance (87.69 ± 5.69 vs. 84.15 ± 8.35, *P* = 0.018), stronger service-learning abilities (288.76 ± 47.28 vs. 259.66 ± 46.51, *P* = 0.003), and more positive course evaluation scores (37.84 ± 3.85 vs. 34.81 ± 4.88, *P* = 0.001). Qualitative findings further showed that the intervention supported knowledge application in practice, strengthened communication and caring, and deepened students' understanding of the value of TCM nursing. Students also reported increased workload and time demands.

**Conclusion:**

Service-learning using the BOPPPS model may be a valuable teaching approach in TCM nursing education. It may help students connect classroom learning with authentic care needs while fostering practical competence and humanistic awareness.

## 1 Introduction

Nursing education is increasingly emphasizing learning approaches that extend beyond knowledge acquisition to include experiential learning and the development of professional values (1). To respond effectively to the complex needs of contemporary healthcare systems, future nurses are expected not only to demonstrate clinical competence but also to cultivate service awareness, humanistic care, and social responsibility (2, 3). In this context, service-learning has been increasingly recognized as a valuable educational approach in nursing education. As a course-based pedagogy, it integrates meaningful community service with academic learning and structured reflection (4, 5). Unlike general volunteer service, service-learning goes beyond participation in service activities by emphasizing learning through service and growth through reflection (6). In nursing education, it has been widely used in community and public health nursing, gerontological nursing, and courses emphasizing leadership, social responsibility, or community engagement (7–9). Previous studies have shown that service-learning can promote knowledge application, communication, teamwork, empathy, and professional responsibility (10).

These educational goals are highly consistent with the aims of traditional Chinese medicine (TCM) nursing education. TCM nursing is a practice-oriented field of nursing grounded in TCM theory. It emphasizes holism and nursing care based on syndrome differentiation and is widely applied in disease prevention, health promotion, clinical care, and rehabilitation (11, 12). TCM nursing education is characterized by strong practical relevance and a distinct humanistic orientation. In addition to acquiring theoretical knowledge and technical skills, students are expected to develop holistic nursing thinking, patient-centered caring awareness, and an understanding of the social value of nursing practice in authentic contexts (13). However, current TCM nursing teaching still relies largely on classroom-based instruction and campus-based skills training, with limited opportunities for students to engage with authentic service recipients or real practice contexts (14). Although these approaches are useful for knowledge transmission and skills training, they may be less effective in helping students understand authentic health needs, develop context-based caring abilities, and engage in reflective learning (15). These limitations highlight the need for teaching strategies that better connect classroom learning with authentic service contexts. In this regard, service-learning appears to offer a meaningful pedagogical approach for TCM nursing education.

Although service-learning often involves processes such as preparation, service, reflection, and celebration, its educational value in formal coursework still depends on clear instructional design and structured pedagogical support (7). The BOPPPS model is a well-established instructional framework consisting of six components: bridge-in, objectives, pre-assessment, participatory learning, post-assessment, and summary (16). By providing a structured sequence for teaching and learning, it may offer useful support for organizing service-learning in a more coherent and educationally purposeful way. Previous work has applied the BOPPPS model to theoretical teaching in TCM nursing and reported relevant experience in teaching reform (17). However, the use of the BOPPPS model to structure service-learning in TCM nursing education remains insufficiently explored. Therefore, the present study integrated the BOPPPS model into service-learning in TCM nursing education and used a mixed-methods design to examine its educational effects and explore students' learning experiences.

## 2 Methods

### 2.1 Study design

This study used a mixed-methods design to examine the educational effects of service-learning using the BOPPPS model in TCM nursing education. The quantitative component adopted a quasi-experimental design with between-group comparisons to evaluate the effects of the intervention. The qualitative component analyzed students' final reflective reports using thematic analysis to further understand their learning experiences and perceptions of the intervention.

### 2.2 Participants

The target population comprised undergraduate nursing students receiving TCM nursing education. This study was conducted at a traditional Chinese medicine university in China from February 1 to June 30, 2025. The undergraduate nursing program consists of four academic years and eight semesters, and *TCM Nursing* is offered as a compulsory course in the second semester. The study included 96 undergraduate nursing students from two intact classes enrolled in this course. The sample size was determined by the total number of students in these two classes, rather than by an *a priori* sample size calculation. These classes were formed by the university upon enrollment through an administrative random allocation system and were not specifically constituted for this study. To minimize disruption to the existing teaching arrangements, the two intact classes were assigned to the experimental group (*n* = 49) or the control group (*n* = 47) at the class level using a random number generator. All participants had completed foundational TCM courses in the first semester, including *Basic Theories of TCM* and *Diagnostics of TCM*, which provided a comparable knowledge base prior to the intervention.

### 2.3 Course arrangement

The course lasted for 16 weeks and consisted of one 90-minute theoretical session each week and one 180-minute practical session every two weeks. The theoretical instruction was delivered by the same instructor to both the experimental and control groups using standardized teaching materials and instructional procedures. These sessions focused on core theoretical knowledge related to TCM nursing. The practical component covered three main areas: (1) fundamental TCM nursing skills, such as daily living care, emotional care, medication care, and health education; (2) health preservation knowledge, including TCM constitution assessment, seasonal health management, and traditional exercise therapies; and (3) traditional TCM nursing techniques, including cupping, gua sha, moxibustion, acupoint massage, and auricular acupressure. Before their respective practical sessions, both groups were provided with the same course-related learning materials through the online course platform. The control group followed the standard curriculum and completed all practical modules through conventional teaching methods. Instructors conducted demonstrations in the on-campus skills laboratory, followed by small-group practice and in-lab simulations based on clinical cases. In contrast, the experimental group completed the practical component through service-learning activities in community settings using the BOPPPS model.

#### 2.3.1 Service-learning projects

The service-learning projects were designed to provide students with opportunities to apply theoretical knowledge of TCM nursing in authentic community settings. Before the course began, a teaching and research team was established, and consultations with clinical TCM nursing experts were conducted to determine the final set of projects (Table 1). The community partners were local community health service centers with which the university had established collaborative relationships, and the direct recipients of the services were community residents. The course instructors communicated the project objectives, activity content, and implementation plans to the administrators of these centers. The instructors and center staff then jointly coordinated the activity schedules, venues, and other practical arrangements, while center staff assisted in inviting residents to participate. During the course, the experimental group participated in structured activities at these centers every two weeks, focusing on health promotion and TCM-based nursing interventions. Students were organized into small groups of 4–6 members and participated in all seven projects, with flexibility to tailor specific content to the needs of different groups of community residents. For example, in the TCM Exercise Workshop, some groups selected Tai Chi, whereas others practiced Baduanjin. Faculty members provided ongoing guidance through pre-visit briefings, on-site supervision, and post-activity debriefings. Students documented their experiences and reflections in learning logs, which were later discussed in group meetings to facilitate reflection and consolidate learning.

**Table 1:** Overview of service-learning projects in the TCM nursing course.

No.	Week	Project Title	Target Group	Service Content	Targeted Knowledge and Skills
1	Week 2	TCM Herbal Sachet Workshop	Schoolchildren in the Community	Organize interactive TCM-themed games and guide children in making TCM sachets.	Chinese herbal knowledge; manual skills; cultural communication.
2	Week 4	Seasonal TCM Diet Workshop	Community residents	Guide community residents in preparing and tasting seasonal medicated diets while providing health education on dietary therapy.	Seasonal health preservation; dietary therapy; contraindications.
3	Week 6	TCM Exercise Workshop	Community-dwelling older adults	Demonstrate and guide community older adults in traditional Chinese exercises, including Baduanjin, Wuqinxi, and Tai Chi.	Theoretical basis of traditional exercises; movement principles; safety assessment; instructional skills.
4	Week 8	TCM Culture and Health Promotion Poster Project	Community residents	Design and present posters on TCM culture and health knowledge.	Health education; communication skills; teamwork; creative presentation.
5	Week 10	Parent–Child Pediatric Tuina Workshop	Parents of young children in the community	Demonstrate pediatric tuina techniques for common childhood conditions, such as cough and diarrhea.	Meridians and acupoints; common pediatric conditions; pediatric tuina skills.
6	Week 12	Practical TCM Nursing Techniques Workshop	Community residents with suboptimal health status	Based on TCM assessment, apply techniques such as gua sha, moxibustion, and cupping to support residents' health management.	Four diagnostic methods of TCM; meridians and acupoints; TCM nursing techniques.
7	Week 14	Home-based TCM Nursing Service Project	Community-dwelling older adults living alone	Measure service recipients' vital signs, assess their TCM constitution, and provide lifestyle guidance.	Syndrome differentiation-based nursing; elderly care; communication skills.

#### 2.3.2 Design of service-learning projects using the BOPPPS model

**Figure 1:**
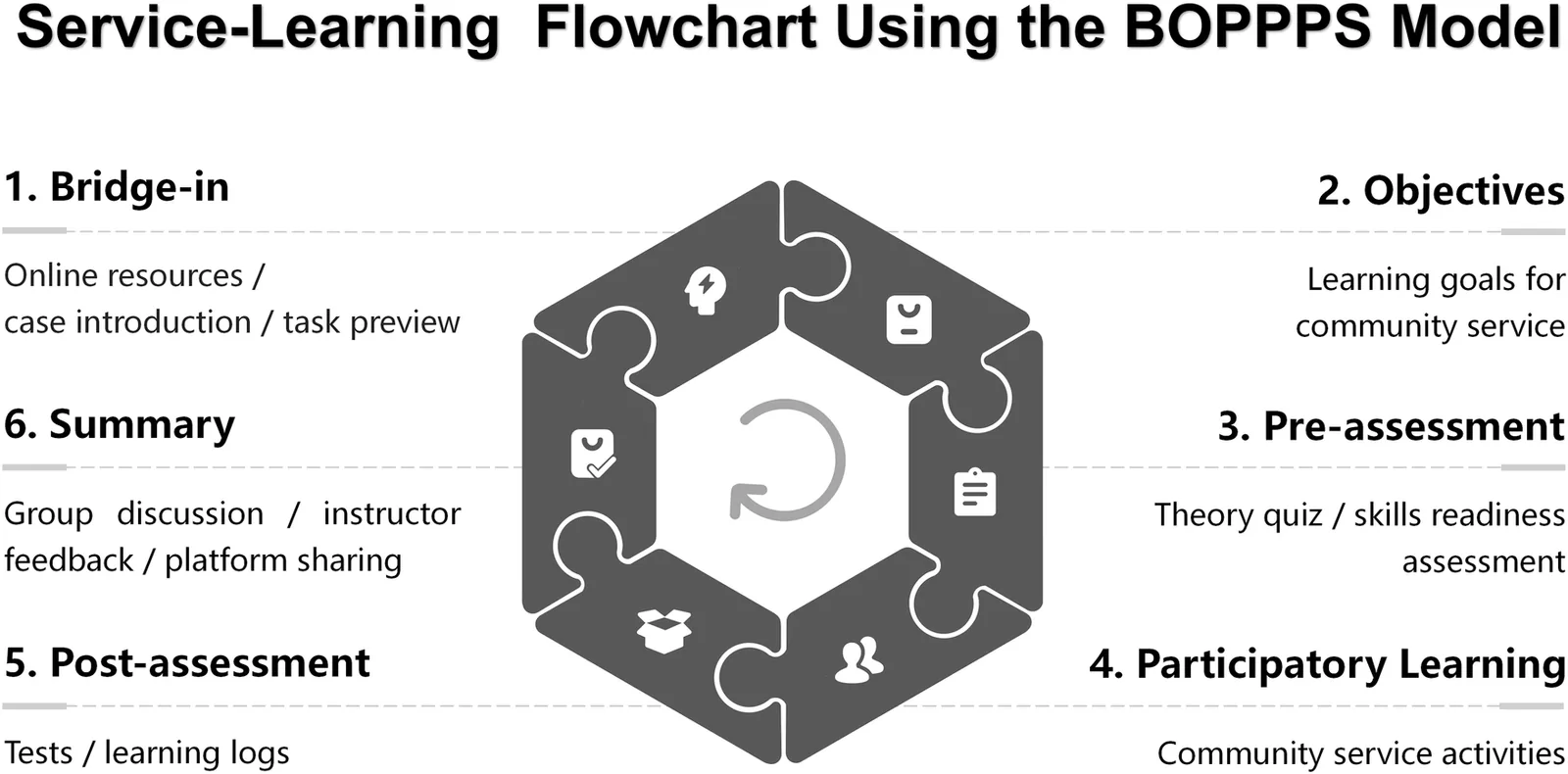
Service-learning implementation flowchart using the BOPPPS model in the *TCM nursing* course.

The service-learning activities were designed and implemented according to the six components of the BOPPPS model: bridge-in, objectives, pre-assessment, participatory learning, post-assessment, and summary. It emphasizes active student participation and ongoing feedback throughout the learning process. In this study, the BOPPPS model was used to guide the design and implementation of service-learning activities, so that the course was delivered in a structured and student-centered manner. Figure 1 illustrates the overall implementation flow of the service-learning projects designed using the BOPPPS model. The TCM Exercise Workshop is presented here as an illustrative example.

##### Bridge-in

Two weeks before the activity, instructors uploaded videos, traditional stories, and health cases related to traditional Chinese exercises to the course online platform. These materials introduced Tai Chi, Baduanjin, and Wuqinxi (Five-Animal Exercise) and prepared students for the upcoming community activity.

##### Objectives

Based on the syllabus and activity content, instructors specified the learning objectives. Students were expected to understand the theoretical basis and key principles of traditional Chinese exercises, conduct safety assessment for older adults, and appropriately demonstrate and guide participants during practice.

##### Pre-assessment

Before the community activity, students completed a pre-assessment consisting of theoretical tests (e.g., exercise safety for older adults and the relationship between Zang-Fu organs and respiratory regulation) and practical evaluation through on-campus simulation of selected exercise movements. This step was used to assess students' readiness for community teaching.

##### Participatory learning

Students worked in small groups to deliver exercise demonstrations and guidance to older adults in the community. Each group selected appropriate exercises according to participants' health conditions, while instructors provided on-site supervision.

##### Post-assessment

After the activity, students completed online tests to assess their knowledge of traditional Chinese exercises, and submitted learning logs to document their reflections on the activity.

##### Summary

Students shared their experiences and reflections in group discussions, and instructors provided feedback. Selected reflections from the learning logs were then posted on the course online platform for class-wide sharing.

### 2.4 Assessment of teaching outcomes

Teaching outcomes were assessed using academic performance, the Service-learning Abilities Scale, the Course Evaluation Questionnaire, and qualitative reflective reports. The two questionnaires were administered to students in the experimental and control groups through the online course platform at the end of the course, after the teaching activities in both groups had been completed.

#### 2.4.1 Academic performance

Academic performance was assessed using both formative and summative components, with a total score of 100 points. The formative assessment accounted for 40% of the total score and included three components: practical training (20%), which evaluated students' performance in practical sessions; self-directed learning (15%), which was based on assignments and online learning engagement; and attendance (5%), for which deductions were applied only in cases of unexcused absence, whereas approved sick leave or personal leave did not affect the score. At the end of the semester, both groups completed a closed-book examination to assess their understanding of fundamental TCM nursing knowledge. This summative assessment accounted for 60% of the total score.

#### 2.4.2 Service-learning abilities scale

The Service-learning Abilities Scale used in this study was originally developed and psychometrically validated by Chao et al. for use in Asian populations (18). The instrument consists of 36 items across nine dimensions, with four items in each dimension: (1) self-knowledge and self-confidence, (2) communication skills, (3) problem-solving skills, (4) citizenship and social responsibility, (5) team skills, (6) self-reflection, (7) knowledge application, (8) caring for others, and (9) cross-cultural competence. Each item is scored from 0 (strongly disagree) to 10 (strongly agree), with higher total scores indicating stronger service-learning abilities. Previously reported Cronbach's *α* coefficients ranged from 0.86 to 0.93. In the present study, Cronbach's *α* coefficients for the subscales ranged from 0.82 to 0.94, indicating acceptable to excellent internal consistency. The full list of items for the Service-learning Abilities Scale is provided in Appendix 1.

#### 2.4.3 Course evaluation questionnaire

The Course Evaluation Questionnaire was developed by the teaching team based on the course objectives and had previously been used in a study of TCM nursing education (17). The questionnaire consisted of eight items covering key aspects of the learning experience, including learning interest, classroom efficiency and content delivery, teamwork and interpersonal communication, information retrieval and literature appraisal, nursing competence in syndrome differentiation, critical thinking and innovation ability, confidence in applying TCM nursing in clinical practice, and interdisciplinary learning and knowledge integration. For the present study, two experts in TCM nursing education who were independent of the research team qualitatively reviewed the items for relevance, clarity, and alignment with the intended course outcomes. The questionnaire was also pilot-tested with 10 undergraduate nursing students who were not included in the main study to assess item clarity and readability. Following the expert review and pilot testing, the original three-category response format used in the previous study was replaced with a 5-point Likert scale, while the eight items remained unchanged. In the final version, responses ranged from 1 (strongly disagree) to 5 (strongly agree), with higher scores indicating more positive evaluations of the course. In the present study, the questionnaire demonstrated good internal consistency, with a Cronbach's *α* coefficient of 0.93. The full questionnaire is provided in Appendix 2.

#### 2.4.4 Qualitative reflective reports

At the end of the course, students in the experimental group were invited to submit a final reflective report describing their overall service-learning experience in TCM nursing. Submission was voluntary, and all reports were anonymized before analysis. No word limit was imposed. Students were encouraged to reflect on their learning experiences, perceived gains, challenges encountered, and any new understanding of TCM nursing gained through the course. Unlike the learning logs completed after each activity, these final reflective reports focused on students' overall experience across the entire course. They served as the qualitative data source for the subsequent analysis.

### 2.5 Data analysis

Quantitative data were analyzed using SPSS version 26.0. Participant characteristics were summarized using descriptive statistics. Continuous variables were presented as mean ± standard deviation and compared using independent-samples t-tests, whereas categorical variables were analyzed using chi-square tests. A two-sided *P* value < 0.05 was considered statistically significant. Effect sizes for between-group differences in academic performance, service-learning abilities, and course evaluation scores were calculated using Cohen's *d*. *Post-hoc* statistical power was calculated using G*Power version 3.1.9.7 based on the observed effect sizes, group sample sizes, and a two-sided significance level of 0.05.

Qualitative data were managed using NVivo version 11.0 and analyzed following Braun and Clarke's six-phase thematic analysis framework (19). First, two researchers (SH and YY) independently read the anonymized reflective reports several times to become familiar with the data. They then systematically coded relevant text segments across the reports, focusing on students' overall service-learning experiences and understanding of TCM nursing. After comparing their initial codes, the two researchers collated related codes to develop preliminary themes. The candidate themes were subsequently reviewed against both the coded extracts and the entire dataset to assess whether they were coherent, distinct, and adequately supported by the data. Through this iterative process, the themes were refined, defined, and named. Finally, the findings were organized into an analytical narrative, and representative quotations were selected to illustrate each theme. SH and YY compared their coding and thematic interpretations throughout the analysis and resolved discrepancies through discussion. When consensus could not be reached, a third researcher (HS) was consulted.

### 2.6 Ethical considerations

The study was approved by the institutional ethics committee (Approval No. AHUCM-HSS-2024012). All procedures involving human participants were conducted in accordance with institutional guidelines and the ethical standards of the relevant national research committee, as well as the 1964 Declaration of Helsinki and its later amendments. Written informed consent was obtained from all participants prior to data collection. Participants were informed that their data would be anonymized and kept confidential, and that all personally identifiable information would be removed.

## 3 Results

### 3.1 Demographic characteristics of the participants

Table 2 presents the demographic characteristics of the two groups. The control group included 19 males and 28 females, with a mean age of 18.91 ± 0.75 years, whereas the experimental group included 17 males and 32 females, with a mean age of 19.02 ± 0.69 years. No statistically significant differences were found between the two groups in terms of gender, age, place of origin, or only-child status (*P* > 0.05), indicating baseline comparability.

**Table 2:** Participant characteristics.

Characteristics	Experimental group	Control group	*χ*^2^/t	*P* value
Gender			0.336	0.562
Male, n (%)	17 (34.69)	19 (40.43)		
Female, n (%)	32 (65.31)	28 (59.57)		
Age in years			0.718	0.474
Mean ± SD	19.02 ± 0.69	18.91 ± 0.75		
Place of origin			0.363	0.547
Urban, n (%)	13 (26.53)	10 (21.28)		
Rural, n (%)	36 (73.47)	37 (78.72)		
Only-child status			1.803	0.179
Yes, n (%)	8 (16.33)	13 (27.66)		
No, n (%)	41 (83.67)	34 (72.34)		

### 3.2 Quantitative outcomes

#### 3.2.1 Comparison of course scores between groups

Table 3 presents the comparison of course scores between the two groups. The experimental group scored significantly higher than the control group in the final examination, practical training, self-directed learning, and total course score (*P* < 0.05).

**Table 3:** Comparison of course scores between groups.

Assessment Component	Experimental group	Control group	*t*	*P* value
Final examination score	80.84 ± 9.22	76.00 ± 13.63	2.028	0.046*
Practical training score	97.78 ± 0.62	95.72 ± 0.65	15.822	<0.001**
Self-directed learning score	97.08 ± 1.29	95.55 ± 2.42	3.838	<0.001**
Total course score	87.69 ± 5.69	84.15 ± 8.35	2.420	0.018*

Attendance scores are not shown because all students received full marks. Values are presented as mean ± SD. *P* < 0.05 was considered statistically significant. *P < 0.05; **P < 0.01.

#### 3.2.2 Comparison of service-learning abilities between groups

As shown in Table 4, after the intervention, the experimental group scored significantly higher than the control group in overall service-learning ability (288.76 ± 47.28 vs. 259.66 ± 46.51, *P* = 0.003) as well as in all nine individual dimensions (all *P* < 0.05). The between-group differences were particularly notable in self-knowledge and self-confidence (*P* = 0.004), citizenship and social responsibility (*P* = 0.004), self-reflection (*P* = 0.001), and cross-cultural competence (*P* = 0.002).

**Table 4:** Comparison of service-learning abilities between groups.

Dimension	Experimental group	Control group	*t*	*P* value
Self-knowledge and self-confidence	31.27 ± 5.56	27.81 ± 5.81	2.980	0.004**
Communication skills	30.55 ± 6.34	27.77 ± 4.64	2.463	0.016*
Problem-solving skills	31.22 ± 6.12	28.15 ± 5.40	2.606	0.011*
Citizenship and social responsibility	33.27 ± 5.61	29.85 ± 5.87	2.913	0.004**
Team skills	32.59 ± 5.79	29.49 ± 6.01	2.577	0.012*
Self-reflection	32.16 ± 5.42	27.85 ± 6.61	3.501	0.001**
Knowledge application	32.37 ± 5.70	29.38 ± 6.78	2.337	0.022*
Caring for others	32.73 ± 5.34	30.28 ± 6.20	2.085	0.040*
Cross-cultural competence	32.59 ± 5.22	29.09 ± 5.68	3.153	0.002**
Total score	288.76 ± 47.28	259.66 ± 46.51	3.038	0.003**

Values are presented as mean ± SD. *P < 0.05; **P < 0.01.

#### 3.2.3 Comparison of course evaluation between groups

As shown in Table 5, the experimental group reported significantly higher overall instructional evaluation than the control group (37.84 ± 3.85 vs. 34.81 ± 4.88, *P* = 0.001). Significant between-group differences were observed in learning interest in TCM nursing, teamwork and interpersonal communication skills, nursing competence in syndrome differentiation, critical thinking and innovation ability, confidence in applying TCM nursing in clinical practice, and interdisciplinary learning and knowledge integration (all *P* < 0.05). No statistically significant differences were found between the groups in classroom efficiency and content delivery or in information retrieval, literature appraisal, and presentation skills (*P* > 0.05).

**Table 5:** Comparison of course evaluation scores between groups.

Item	Experimental group	Control group	*t*	*P* value
1. The course design effectively stimulates interest in TCM nursing.	4.80 ± 0.50	4.34 ± 0.84	3.209	0.002**
2. The course design improves classroom efficiency and content delivery.	4.53 ± 0.65	4.57 ± 0.65	−0.331	0.742
3. The course design fosters teamwork and interpersonal communication skills.	4.76 ± 0.52	4.40 ± 0.77	2.601	0.011*
4. The course design develops skills in information retrieval, literature appraisal, and presentation.	4.71 ± 0.54	4.51 ± 0.69	1.609	0.111
5. The course design facilitates the development of nursing competence in syndrome differentiation.	4.73 ± 0.53	4.38 ± 0.74	2.668	0.009**
6. The course design facilitates the development of critical thinking and innovation ability.	4.82 ± 0.44	4.38 ± 0.71	3.579	0.001**
7. The course design strengthens confidence in applying TCM nursing in clinical practice.	4.73 ± 0.49	4.23 ± 0.89	3.394	0.001**
8. The course design promotes interdisciplinary learning and the integrated application of knowledge.	4.76 ± 0.48	3.98 ± 1.09	4.473	<0.001**
Total score	37.84 ± 3.85	34.81 ± 4.88	3.382	0.001**

Values are presented as mean ± SD. *P < 0.05; **P < 0.01.

Effect size analyses further characterized the magnitude of the between-group differences across the three quantitative outcomes. The largest effect was observed for course evaluation scores (Cohen's *d* = 0.69; *post-hoc* powe*r* = 0.92), followed by service-learning abilities (Cohen's *d* = 0.62; *post-hoc* powe*r* = 0.85) and academic performance (Cohen's *d* = 0.50; *post-hoc* powe*r* = 0.67).

### 3.3 Qualitative findings

A total of 45 final reflective reports (S1–S45), comprising approximately 25,000 words, were collected from students in the experimental group. Thematic analysis generated four main themes: (1) from knowledge to practice in authentic service contexts; (2) communication and caring through real service encounters; (3) deepening understanding of the value of TCM nursing; and (4) increased workload and time demands in service-learning.

#### 3.3.1 From knowledge to practice in authentic service contexts

Students reported that service-learning helped them apply TCM nursing knowledge in authentic community settings. Working with real service recipients made previously abstract content more concrete and meaningful, while also increasing students' awareness of the responsibility involved in practice. Several students noted that this process made their understanding of syndrome differentiation and individualized care more practical, as they needed to adapt their knowledge and skills to recipients' specific needs.

“What I had memorized last semester about tongue diagnosis, pulse diagnosis, and meridian theory suddenly came alive when I used it in the community. “ (S12)

“In classroom practice with peers, mistakes in acupoint selection or excessive pressure could usually be corrected without serious consequences. However, when I provided acupoint massage for an older adult with hypertension in the community, I felt a strong sense of responsibility for the first time. Every amount of pressure and every choice of acupoint required careful consideration rather than routine practice.” (S37)

#### 3.3.2 Communication and caring through real service encounters

Students frequently reported that interaction with real service recipients improved their communication skills and strengthened their awareness of caring in practice. They noted that effective communication required not only explaining TCM knowledge in simpler language, but also listening to recipients' concerns and adjusting guidance accordingly. These experiences helped students realize that building trust and showing empathy were important parts of nursing care.

“One older man was initially unwilling to practice Baduanjin with us because he felt the movements might not help and worried that his body could not manage them. Instead of persuading him right away, I first listened to his concerns and suggested that he start with a few easier movements. Gradually, he joined us and later said that he felt more comfortable after practicing. This made me realize that building trust and understanding recipients' concerns can sometimes be more important than explaining knowledge first.” (S9)

“During the activity, I felt that the greatest difficulty was communicating with older adults. The professional terms we use in class were too difficult for them to understand, so I needed to introduce the content in a much simpler way.” (S18)

#### 3.3.3 Deepening understanding of the value of TCM nursing

Students reported that service-learning deepened their understanding of the value of TCM nursing. Through direct contact with service recipients, they became more aware that what they had learned could be meaningfully applied in practice. Some students also reported a stronger sense of professional value, as they began to view TCM nursing as a practice that involves assessment, guidance, and health management rather than merely technical operation.

“After I used gua sha to help relieve one woman's neck and shoulder discomfort, she held my hand and said, ‘My neck feels much better—you are really skillful.’ The gratitude and relief in her eyes made me truly realize that what I had learned could make a real difference.” (S24)

“I used to think that nursing was mainly about carrying out doctors' orders, and I was not very interested in this major. However, through this semester's learning, I came to realize that, by using the four diagnostic methods of TCM and syndrome differentiation, we can provide systematic guidance on diet, emotional well-being, and daily living. This made me see TCM nursing as more than simple technical work, and more like a form of health management.” (S31)

#### 3.3.4 Increased workload and time demands in service-learning

Students also reported that service-learning required substantial time and effort beyond regular classroom learning. Compared with conventional teaching, service-learning involved more preparation, group coordination, and after-class work, which sometimes increased academic pressure. Several students noted that balancing service-learning tasks with other coursework could be challenging, even though these demands also encouraged them to prepare more thoroughly and engage more deeply with the course content.

“Our group held three meetings to prepare the health education poster, and we stayed up late choosing the topic, searching for information, and designing the poster. Although the activity was very successful, the quality of my assignments for other courses was indeed affected that week. It felt like robbing Peter to pay Paul.” (S15)

“During the Parent–Child Pediatric Tuina Workshop, there were many acupoints involved, and I worried that parents might ask questions I could not answer, which made me feel a lot of pressure. At the same time, the activity also helped me remember this knowledge much more firmly.” (S41)

## 4 Discussion

The findings of this study suggest that service-learning using the BOPPPS model may be a useful pedagogical approach in TCM nursing education. Compared with conventional teaching, it was associated with better academic performance, stronger service-learning abilities, and more positive course evaluations. Among the three quantitative outcomes, the largest standardized between-group difference was observed for course evaluation scores, followed by service-learning abilities and academic performance. Qualitative findings further indicated that the intervention promoted knowledge application in authentic practice, strengthened communication and caring through real service encounters, and deepened students' understanding of the value of TCM nursing. However, students also reported increased workload and time demands associated with service-learning.

The better academic performance observed in the experimental group may be partly explained by the way service-learning connected classroom knowledge with authentic practice. Previous studies have shown that service-learning can strengthen knowledge application and support theory–practice integration in nursing education (6). Qualitative evidence further indicates that learning becomes more concrete and meaningful when students work with real service recipients (20). In the present study, students were required not only to recall TCM nursing knowledge, but also to interpret, adapt, and apply it according to recipients' specific conditions and concerns. In this way, abstract knowledge had to be translated into concrete guidance and care. This process may have contributed to the higher course scores observed in the experimental group, including the final examination, practical training, and self-directed learning components, as well as to stronger service-learning abilities related to knowledge application. This is especially meaningful in TCM nursing education, which places strong emphasis on applying knowledge to individualized care and practical guidance in real-world contexts (21). A similar pattern was also reported in Chinese medicine education, where service-learning supported the teaching of Tui-na by connecting practice-oriented learning with authentic service contexts (22).

The higher scores in communication, caring, and teamwork-related outcomes also deserve attention. These dimensions are increasingly regarded as important components of nursing education alongside disciplinary knowledge and technical skills (23, 24). Existing evidence suggests that service-learning can foster such development by placing students in real interactions that require them to respond to recipients' concerns, emotions, and feedback in real time (25). This may be especially relevant to TCM nursing education. Its humanistic orientation, rooted in the traditional ideal of benevolence in medicine, places emphasis on sympathy, respect, patient communication, and emotional support in addition to technical care (26). In the present study, students did not simply participate in activities. They were required to explain TCM nursing knowledge in accessible language, listen carefully to recipients' concerns, build trust, and coordinate with peers to complete shared tasks. Through this process, communication became a relational and caring practice rather than a one-way delivery of information, while teamwork developed through shared planning, coordination, and problem-solving. These experiences may help explain the higher service-learning abilities observed in communication, caring, and teamwork-related domains, and may also have contributed to more positive evaluations of the course. Reflection after service activities may have further reinforced this process by helping students review their interactions and deepen their understanding of the humanistic values embedded in TCM nursing (27).

At a broader level, service-learning appeared to reshape how students understood both the TCM nursing course and the nursing profession itself. By engaging in authentic community service, students were no longer merely passive recipients of knowledge, but gradually became service providers capable of responding to real needs and addressing practical problems. This shift in role created a more direct connection between course learning and real care contexts, and also enabled students to explore and clarify their future professional direction through practice (28). At the same time, overcoming challenges during service, perceiving recipients' feedback, and gaining a sense of achievement and confidence appeared to strengthen students' perceptions of the value of the course and the significance of the profession (29). This is meaningful because professional identity in nursing involves not only emotional attachment to the profession, but also an integrated understanding of professional values, knowledge systems, and occupational roles. Previous studies have shown that a clear professional identity can promote learning engagement and academic achievement (30, 31), and is also closely associated with empathy and other humanistic capacities (32). In this study, such changes may help explain the more positive overall course evaluations in the experimental group, particularly in relation to learning interest, practical relevance, and the perceived value of the course. Taken together with the qualitative findings, these results suggest that service-learning may enhance students' perceived course value and support the development of professional identification by helping them view TCM nursing as a meaningful and applicable form of care in real service contexts, rather than as a set of isolated knowledge points or technical requirements.

While the above findings may be explained by authentic service experiences themselves, another important consideration is how those experiences were pedagogically structured. An additional factor that may help explain the positive outcomes observed in this study is the structured instructional support provided by the BOPPPS model. Previous evidence in nursing education suggests that BOPPPS, as a student-centered and closed-loop teaching model, is associated with better theoretical and practical performance, stronger autonomous learning, and higher learning satisfaction than conventional teaching (16, 33). More recent studies have further shown that this approach can promote professional identity, academic self-efficacy, and learning engagement in undergraduate nursing education (34, 35). In the present study, service-learning was not implemented as an isolated community activity, but was embedded within a teaching framework aligned with course objectives, learner preparation, service practice, assessment and feedback, and reflective summary. Such a structured design may have helped maintain a close connection between service activities and course learning, making students' practical experiences more likely to develop into observable and assessable learning outcomes. In particular, explicit objectives helped ensure that service activities remained aligned with course requirements, assessment and feedback enabled students to identify problems and adjust their learning strategies in a timely manner, and summary and reflection further supported the organization, understanding, and internalization of experience (36). Accordingly, the positive changes observed in the experimental group may reflect not only the value of authentic service contexts, but also the structured way in which service-learning was designed and guided.

Despite the generally positive outcomes, the implementation of service-learning also entailed certain practical costs. The experimental group achieved a significantly higher overall course evaluation score than the control group. However, no significant advantage was observed for the item related to classroom efficiency and content delivery, and the experimental group even scored slightly lower on this item. Considered alongside the qualitative findings, this may be related to the greater demands of service-learning in terms of pre-class preparation, teamwork, real-time adaptation in authentic settings, and post-activity reflection. These findings suggest that service-learning is not a low-input teaching approach, but rather a course design that places relatively high demands on both student engagement and teacher organization (37). Future implementation should therefore focus on optimizing activity arrangements, reasonably managing workload, and providing more adequate instructional support in order to achieve a better balance between learning gains and implementation costs.

## 5 Limitations

Several limitations of this study should be acknowledged. First, this was a single-center study with a relatively modest sample size, which may limit statistical precision and generalizability of the findings to other institutions and educational contexts. Second, the study adopted a quasi-experimental design, and potential confounding factors could not be entirely ruled out. Third, some quantitative outcomes were based on self-reported scales and may therefore be subject to response bias, while the qualitative data were derived from students' final reflective reports rather than interviews, which may not have fully captured the complexity of their experiences (38). Fourth, this study focused on short-term outcomes assessed at the end of the course, and no follow-up was conducted to determine whether the observed effects could be sustained over time. Finally, the control group was not subsequently offered the BOPPPS-based service-learning intervention due to the limited time available within the scheduled course period, although they received the full standard curriculum required by the university. Future research should include larger, multi-center samples, more rigorous study designs, and longitudinal assessment of educational outcomes.

## 6 Conclusion

This study suggests that service-learning using the BOPPPS model may be a useful pedagogical approach in TCM nursing education. By combining structured instructional design with authentic community service, it may help students connect classroom learning with real care needs and deepen their understanding of the practical and humanistic value of TCM nursing. Therefore, structured service-learning may be a promising strategy for fostering practical competence and humanistic awareness in undergraduate TCM nursing education.

## Data Availability

The raw data supporting the conclusions of this article will be made available by the authors, without undue reservation.

## References

[1] Sumpter D Blodgett N Beard K Howard V. Transforming nursing education in response to the future of nursing 2020–2030 report. Nurs Outlook. (2022) 70(6 Suppl 1):S20–31. 10.1016/j.outlook.2022.02.007 36446537

[2] World Health Organization. State of the World's Nursing 2025: Investing in Education, Jobs and Leadership. Geneva: World Health Organization (2025). Available online at: https://www.who.int/publications/i/item/9789240110236 (Accessed March 20, 2026).

[3] Zhang J Tian Y. Final-year nursing students' Perceptions of humanistic education in nursing: a cross-sectional descriptive study. BMC Med Educ. (2024) 24(1):392. 10.1186/s12909-024-05377-3 38594668 PMC11005204

[4] Altaker KL. Service learning in an evidence-based practice course. Teach Learn Nurs. (2024) 19(3):e467–70. 10.1016/j.teln.2024.02.001

[5] Ratnayake A Lederer A. Service learning in public health: a critical assessment of potential benefits and unintended consequences. Pedagogy Health Promot. (2024) 10(1):11–5. 10.1177/23733799231177043

[6] Marcilla-Toribio I Moratalla-Cebrián ML Bartolomé-Gutiérrez R Cebada-Sánchez S Galán-Moya EM Martínez-Andrés M. Impact of service-learning educational interventions on nursing students: an integrative review. Nurse Educ Today. (2022) 116:105417. 10.1016/j.nedt.2022.105417 35691112

[7] Yoong SQ Liao AWX Goh SH Zhang H. Educational effects of community service-learning involving older adults in nursing education: an integrative review. Nurse Educ Today. (2022) 113:105376. 10.1016/j.nedt.2022.105376 35489329

[8] Emrani M Khoshnood Z Farokhzadian J Sadeghi M. The effect of service-based learning on health education competencies of students in community health nursing internships. BMC Nurs. (2024) 23(1):138. 10.1186/s12912-024-01799-y 38395792 PMC10893737

[9] Gresh A LaFave S Thamilselvan V Batchelder A Mermer J Jacques K. Service-learning in public health nursing education: how COVID-19 accelerated community-academic partnership. Public Health Nurs. (2021) 38(2):248–57. 10.1111/phn.12796 32876353

[10] Zhu Z Xing W Liang Y Hong L Hu Y. Nursing students' Experiences with service learning: a qualitative systematic review and meta-synthesis. Nurse Educ Today. (2022) 108:105206. 10.1016/j.nedt.2021.105206 34773814

[11] Hao Y Liu H Yue S Liu X. Introducing traditional Chinese nursing: a review of concepts, theories and practices. Int Nurs Rev. (2011) 58(3):319–27. 10.1111/j.1466-7657.2011.00918.x 21848777

[12] Hao Y Jiang J Gu X. Traditional Chinese medicine and nursing care. Int J Nurs Sci. (2017) 4(3):328–9. 10.1016/j.ijnss.2017.06.005 31406761 PMC6626168

[13] Zhang H Qin Q Hu K Yuan Y Hao S Shi X. Application of narrative medicine in TCM nursing education. Clin Teach. (2025) 22(4):e70110. 10.1111/tct.70110 40500970

[14] Chen L Jiang WJ Zhao RP. Application effect of Kolb's Experiential learning theory in clinical nursing teaching of traditional Chinese medicine. Digit Health. (2022) 8:20552076221138313. 10.1177/20552076221138313 36406155 PMC9669681

[15] Zhou F Yuan T Li Z Mu X Lv Y. The evidence-based practice teaching competence of clinical preceptors at different stages of innovation-decision process: a cross-sectional survey in traditional Chinese medicine hospitals. Nurse Educ Today. (2024) 132:106027. 10.1016/j.nedt.2023.106027 37956570

[16] Zhu J Xiao H Zhou R Gan X Gou Q Tie H. The efficacy of the BOPPPS teaching model in clinical and health education: a systematic review and meta-analysis. BMC Med Educ. (2025) 25(1):997. 10.1186/s12909-025-07274-9 40604993 PMC12224838

[17] Hao S Yuan Y Chen D Shi H Xia Z Zhang C. Application of the BOPPPS model based on chaoxing learning platform in the course fundamentals of traditional Chinese nursing. J Tradit Chin Med Educ. (2023) 42(3):112–6. in Chinese. 10.3969/j.issn.1003-305X.2023.03.734

[18] Chao KY Liu LM Ma CHK Liu HY. Developing and validating the service-learning outcome questionnaire. J Serv Learn Soc Engagem. (2018) 1(1):19–38. in Chinese. 10.6865/JSLSE.201804_(1).0004

[19] Braun V Clarke V. Using thematic analysis in psychology. Qual Res Psychol. (2006) 3(2):77–101. 10.1191/1478088706qp063oa

[20] Saylor J Hertsenberg L McQuillan M O'Connell A Shoe K Calamaro CJ. Effects of a service learning experience on confidence and clinical skills in baccalaureate nursing students. Nurse Educ Today. (2018) 61:43–8. 10.1016/j.nedt.2017.11.009 29169068

[21] Zhou F Lv Y Zhao J. Evidence based practice competence of future traditional Chinese medicine nurses: a cross-sectional online study. Nurse Educ Today. (2022) 110:105238. 10.1016/j.nedt.2021.105238 34999498

[22] Hafiz M Tu F Cheung CH Yue KMK. The impact of e-service-learning on tui-na teaching in a Chinese medicine course—from the perspectives of service-recipients and service-partner. In: Ngai G Shek DT, editors. Service-learning Capacity Enhancement in Hong Kong Higher Education. Singapore: Springer (2022). 14:203–19. 10.1007/978-981-19-2437-8_11

[23] Bassett J Henderson A Baldwin A Frost J. Nurses' Learning about professional interpersonal communication: findings from an integrative review. Nurse Educ Today. (2025) 150:106698. 10.1016/j.nedt.2025.106698 40117720

[24] Badawy W Shaban M. Educational interventions to enhance empathy in undergraduate nursing students: an integrative review. Teach Learn Nurs. (2026) 21(2):182–90. 10.1016/j.teln.2025.08.003

[25] Jing M Chui WH Chong AMC Maoting T. The effects of community-based education programs on empathy, emotional intelligence, and caring behaviors among nursing students: a scoping review. Front Med (Lausanne). (2024) 11:1479466. 10.3389/fmed.2024.1479466 39726680 PMC11669650

[26] Wang L Zhang W Wang Y Wang H Han Y Wang X. Perceived value and educational implications of traditional Chinese medicine nursing: a mixed-methods study. Front Public Health. (2026) 14:1763732. 10.3389/fpubh.2026.1763732 41799444 PMC12963323

[27] Yang Q Zou W. The impact of reflective practice on the development of nursing undergraduate students. BMC Med Educ. (2025) 25(1):880. 10.1186/s12909-025-07490-3 40598300 PMC12211434

[28] Choi Y Han J Kim H. Exploring key service-learning experiences that promote students' Learning in higher education. Asia Pac Educ Rev. (2026) 27:287–302. 10.1007/s12564-023-09833-5

[29] Mumba MN Kaylor SK Townsend H Barron K Andrabi M. Improving confidence in clinical assessment skills through service-learning experiences among nursing students. J Nurs Educ. (2022) 61(5):272–5. 10.3928/01484834-20220303-02 35522760

[30] Zou L Xie Z Tan M Ou Q Liao M. The effect of professional identity on nursing academic achievement: the chain mediating effect of general self-efficacy and learning engagement. BMC Med Educ. (2024) 24(1):1014. 10.1186/s12909-024-05995-x 39285393 PMC11406816

[31] Marold SMG Strouse SM Butcher D. Professional identity in nursing: a narrative review of the ISPIN definition and domains usage. SAGE Open Nurs. (2025) 11:23779608251335240. 10.1177/23779608251335240 40291610 PMC12033407

[32] Amin SM Karim NAHA Osman MA Abdallah HMM El-Sayed AAI Tawfik AF. Exploring the relationship between professional identity, cultural sensibility, and empathy among nursing students: evidence from path analysis. BMC Nurs. (2025) 24(1):628. 10.1186/s12912-025-03235-1 40457416 PMC12131444

[33] Li Y Li S Liu C Li J. Application effect of BOPPPS teaching model on fundamentals of nursing education: a meta-analysis of randomized controlled studies. Front Med (Lausanne). (2024) 11:1319711. 10.3389/fmed.2024.1319711 38784229 PMC11111886

[34] Zhang B Duan A Lan J Ren Y. Application of the BOPPPS teaching model in the teaching of introduction to nursing course. BMC Nurs. (2025) 24(1):1223. 10.1186/s12912-025-03880-6 41023988 PMC12482276

[35] Dai Y He Q Lei L. Effect of the BOPPPS-based blended teaching model in undergraduate nursing education: a quasi-experimental study. Sci Rep. (2026) 16(1):8580. 10.1038/s41598-026-37120-x 41680311 PMC12976344

[36] Staats K Nordhaug M Willassen ET Hansen IN. Learning through reflection in nursing education: perspectives of nursing students. SAGE Open Nurs. (2026) 12:23779608251407794. 10.1177/23779608251407794 41625707 PMC12858777

[37] Lavaux S Salas JI Chiappe A Ramírez-Montoya MS. Regional perspectives on service learning and implementation barriers: a systematic review. Appl Sci. (2025) 15(16):9058. 10.3390/app15169058

[38] DeJonckheere M Vaughn LM. Semistructured interviewing in primary care research: a balance of relationship and rigour. Fam Med Community Health. (2019) 7(2):e000057. 10.1136/fmch-2018-000057 32148704 PMC6910737

