# Supplementary material for: Service-learning using the BOPPPS model in TCM nursing education: a mixed-methods study
Source: Front Med (Lausanne). 2026 Sep 9;13:1859149. doi: 10.3389/fmed.2026.1859149 (PMC13597392; doi:10.3389/fmed.2026.1859149)
Supplement: Supplementary file 1 [file Datasheet1.pdf]

Appendix 1. Service-learning Abilities Scale.

Instructions: Please indicate the extent to which you agree with each statement based on your learning experience in this course by ticking one response option for each item.
Response scale: 0 = strongly disagree, 10 = strongly agree.

Items	Level of Agreement (0–10)
**Dimension 1. Self-knowledge and self-confidence**	
1. I am aware of my strong points and my weak points.	0 1 2 3 4 5 6 7 8 9 10
2. I can keep an open mind, learn from new experiences, and accept challenges.	0 1 2 3 4 5 6 7 8 9 10
3. I often seek out challenging opportunities to examine my skills and abilities.	0 1 2 3 4 5 6 7 8 9 10
4. I am completely confident in my abilities.	0 1 2 3 4 5 6 7 8 9 10
**Dimension 2. Communication skills**	
5. I feel comfortable/at ease expressing my thoughts in front of others.	0 1 2 3 4 5 6 7 8 9 10
6. I know how to communicate my thoughts with others in an unfamiliar situation.	0 1 2 3 4 5 6 7 8 9 10
7. I understand that it is important to communicate with others in a group.	0 1 2 3 4 5 6 7 8 9 10
8. I feel confident that I can correctly communicate my thoughts/opinions/ideas to others.	0 1 2 3 4 5 6 7 8 9 10
**Dimension 3. Problem-solving skills**	
9. I am/feel confident in recognizing problems.	0 1 2 3 4 5 6 7 8 9 10
10. I am/feel confident in solving with problems.	0 1 2 3 4 5 6 7 8 9 10
11. I try my best to collect information about problems before I attempt to solve them.	0 1 2 3 4 5 6 7 8 9 10
12. If I fail to solve a problem on my first attempt, I go back and think about the problem-solving process again.	0 1 2 3 4 5 6 7 8 9 10
**Dimension 4. Citizenship and social responsibility**	
13. I can figure out the important needs of the community or the person who needs help.	0 1 2 3 4 5 6 7 8 9 10
14. I try to be a person who is positive and who can correctly influence communities or people who need help.	0 1 2 3 4 5 6 7 8 9 10
15. I believe that everyone has an obligation to contribute to the community and to individuals needs help.	0 1 2 3 4 5 6 7 8 9 10
16. I have the responsibility to improve the situation if my community or an individual needs help.	0 1 2 3 4 5 6 7 8 9 10
**Dimension 5. Team skills**	
17. When conflict arises in the group, I usually remain reasonable and calm.	0 1 2 3 4 5 6 7 8 9 10
18. I can successfully work with other classmates under various circumstances.	0 1 2 3 4 5 6 7 8 9 10

Items	Level of Agreement (0–10)
19. I can recognize and appreciate the outstanding achievements of others.	0 1 2 3 4 5 6 7 8 9 10
20. I can participate effectively in group discussions and activities.	0 1 2 3 4 5 6 7 8 9 10
**Dimension 6. Self-reflection**	
21. I am confident and independent.	0 1 2 3 4 5 6 7 8 9 10
22. At school, I am motivated to learn, and I participate in/take part in and complete assignments.	0 1 2 3 4 5 6 7 8 9 10
23. I believe that introspection makes me a better person.	0 1 2 3 4 5 6 7 8 9 10
24. When I have completed an assignment, I evaluate myself.	0 1 2 3 4 5 6 7 8 9 10
**Dimension 7. Knowledge application**	
25. I can understand that, in class, learning outcomes and evaluation are important.	0 1 2 3 4 5 6 7 8 9 10
26. I feel confident about using knowledge in a professional field.	0 1 2 3 4 5 6 7 8 9 10
27. I know applying theoretical knowledge in different real-world situations will require adjustments.	0 1 2 3 4 5 6 7 8 9 10
28. When I integrate professional knowledge into reality, my learning is more effective.	0 1 2 3 4 5 6 7 8 9 10
**Dimension 8. Caring for others**	
29. I can understand others' thoughts and feelings.	0 1 2 3 4 5 6 7 8 9 10
30. I believe that, when people have no more bias, this world will be a better place.	0 1 2 3 4 5 6 7 8 9 10
31. When I spend time with people from different backgrounds, I feel comfortable.	0 1 2 3 4 5 6 7 8 9 10
32. I believe that taking care of the people who need help/assistance in society is everyone's responsibility.	0 1 2 3 4 5 6 7 8 9 10
**Dimension 9. Cross-cultural competence**	
33. I love learning about people from different cultural backgrounds.	0 1 2 3 4 5 6 7 8 9 10
34. When I interact with people from different cultural backgrounds, I try to understand their beliefs, perspectives, and feelings.	0 1 2 3 4 5 6 7 8 9 10
35. I believe that if I can notice the differences in body language of people from different cultural backgrounds, it helps me to understand them better.	0 1 2 3 4 5 6 7 8 9 10
36. I am not interested in making friends with people from different cultural backgrounds.	0 1 2 3 4 5 6 7 8 9 10

Appendix 2. Course Evaluation Questionnaire

Instructions: Please indicate the extent to which you agree with each statement based on your learning experience in this course.
Response scale: 1 = strongly disagree, 5 = strongly agree.

Items	Level of Agreement (1–5)
1.The course design effectively stimulates interest in TCM nursing.	1 2 3 4 5
2.The course design improves classroom efficiency and content delivery.	1 2 3 4 5
3.The course design fosters teamwork and interpersonal communication skills.	1 2 3 4 5
4.The course design develops skills in information retrieval, literature appraisal, and presentation.	1 2 3 4 5
5.The course design facilitates the development of nursing competence in syndrome differentiation.	1 2 3 4 5
6.The course design facilitates the development of critical thinking and innovation ability.	1 2 3 4 5
7.The course design strengthens confidence in applying TCM nursing in clinical practice.	1 2 3 4 5
8.The course design promotes interdisciplinary learning and the integrated application of knowledge.	1 2 3 4 5
